# Popular Instruction

**Published:** 1859-11

**Authors:** 


### Editorial.

### POPULAR INSTRUCTION.

The education of the people in regard to any profession, is one very efficient method of elevating that profession. This has been recognized by all the better members of the dental profession, as a very important matter. Let any person understand the true value of the teeth, the necessity for their preservation, and the means by which that can be the most effectually accomplished ; the difference between good and bad operations, and the means by which that may be determined, and dental quackery will soon be at an end ; inferior operations will not be tolerated, a high order of operations will be demanded, and they must be produced. Now what would be true in the case of one person, in this respect, would be true of an hundred, or of a whole community, under similar circumstances. Though the existence of this requirement is so fully acknowledged, yet the best method of meeting the want calls forth some diversity of opinion.

Some suppose they can enlighten their patients more rapidly and more perfectly by conversation than by any other method. Others think that by the judicious employment of well prepared essays among their patients, more can be done than by any other means, and with a great economy of time. The essay containing the proper and desirable information may be referred to by the patient time and again, until its teachings are all perfectly impressed upon the mind ; and when this is the case, they will be carried into practice by the person, just as circumstances may enable him to do it, while that which is simply narrated to the patient may soon be forgotten, and can not be referred to again. And again, too many patients will suspect that there are sinister motives behind the oral explanations and teachings, while nothing of the kind can arise in the mind while perusing a properly prepared essay. Various essays have been prepared for gratuitous distribution, most of them by practitioners for their own use ; some of them, we have observed, partake too much of the advertisement features, and so defeat the true aim of their publication. Some very good works of this kind have been published and put into the market, but the profession, as a general thing, do not seem disposed to take hold of them ; and probably for two or three reasons. In the first place, they cost something, and they have some doubts as to whether they are just the thing that will suit them.

Now, we propose to commence at once the publication of a series of articles in the Register, with the view of giving information upon dental science for the general reader. They will be short and comprehensive, each article discussing but one or two subjects.

The object will be that practitioners may have them copied into the papers or publications of their respective localities, and in that way brought to the notice of their patients and all the people in their respective communities. This can be done without any cost to the practitioner, except the subscription price of the Register.

Now, it is the business of the members of the profession, who feel the want of such articles, to prepare them and send them on to us for publication. We do not intend to prepare more than our quota of these articles, but we do expect the profession to be generous in the supply. Each one might suppose it would answer quite as well to publish his articles at once in his village paper, without publication in the Register at all ; but in that case, his article would not accomplish a tithe of the good it would do if published in two or three hundred papers through the country, and read by ten thousand persons for every hundred that would read it in the former case, and besides, each practitioner could avail himself of all the essays thus published in the Register. The articles when copied into any papers should always bear the name of the authors.

We propose to begin this series immediately, and we hope the members of the profession will prepare and forward to us as soon as possible. Each writer will, of course, choose his subject.

